# The Pathophysiology and Vascular Complications of Diabetes in Chronic Kidney Disease: A Comprehensive Review

**DOI:** 10.7759/cureus.76498

**Published:** 2024-12-28

**Authors:** Wilhelmina N Hauwanga, Tibyan Y Abdalhamed, Lynda A Ezike, Ifeoma S Chukwulebe, Aung Ko Oo, Amal Wilfred, Abdul Rahman Anuz Khan A Khan, Johnny Chukwuwike, Edisond FLORIAL, Habeebah Lawan, Asaju Felix, Billy McBenedict

**Affiliations:** 1 Cardiology, Gaffrée and Guinle University Hospital, Federal University of the State of Rio de Janeiro, Rio de Janeiro, BRA; 2 Neurosurgery, Federal Fluminense University, Niterói, BRA; 3 General Medicine, Garki Hospital, Garki, NGA; 4 Internal Medicine, University of Nigeria, Enugu, NGA; 5 Medicine, Monash University, Melbourne, AUS; 6 General Medicine, Hôpital Sainte Thérèse de Hinche, Port-au-Prince, HTI; 7 General Practice, Dorset County Hospital, Dorchester, GBR

**Keywords:** chronic kidney disease, diabetic nephropathy, inflammation, oxidative stress, type 2 diabetes, vascular complications

## Abstract

The coexistence of type 2 diabetes mellitus (T2DM) and chronic kidney disease (CKD) represents a significant global health challenge, contributing to substantial morbidity, mortality, and economic burden. T2DM is the leading cause of CKD, and CKD exacerbates diabetes-related complications, creating a bidirectional relationship driven by oxidative stress, inflammation, and endothelial dysfunction. Diabetic kidney disease (DKD), affecting some individuals with T2DM, accelerates progression to end-stage renal disease (ESRD) and increases cardiovascular mortality. Microvascular complications, including nephropathy, retinopathy, and neuropathy, and macrovascular complications, such as coronary artery disease and stroke, are prevalent in this population, further diminishing the quality of life.

The pathophysiology underlying these complications is multifaceted. Hyperglycemia-induced oxidative stress and inflammation drive kidney damage and systemic vascular complications, while CKD alters glucose metabolism and antidiabetic drug pharmacokinetics. Endothelial dysfunction exacerbates vascular complications through impaired nitric oxide production and heightened thrombogenicity. Emerging insights into genetic and epigenetic mechanisms, including DNA methylation and mitochondrial dysfunction, have highlighted new therapeutic targets. Management strategies emphasize early screening, glycemic control, and a multidisciplinary approach integrating lifestyle modifications, pharmacotherapy, and patient education. Interventions targeting oxidative stress, inflammation, and endothelial dysfunction have shown promise in mitigating disease progression. Current evidence on the interconnected mechanisms driving DKD and associated vascular complications highlights the critical need for proactive, patient-centered management and further research into innovative diagnostic and therapeutic approaches to address this global health challenge.

## Introduction and background

Type 2 diabetes mellitus (T2DM) is a major public health concern affecting over 460 million individuals globally, and its prevalence is expected to rise due to factors such as aging populations, urbanization, and sedentary lifestyles [1]. The global diabetes prevalence is projected to rise to 10.2% (578 million) by 2030 and 10.9% (700 million) by 2045. The prevalence is higher in urban (10.8%) than rural (7.2%) areas and in high-income (10.4%) than in low-income countries (4.0%) [2,3].

Diabetes imposes a substantial economic burden globally, as evidenced by its direct and indirect costs. In 2002, the estimated total cost of diabetes in the U.S. was $132 billion, comprising $91.8 billion in direct medical expenditures and $40 billion in indirect costs due to lost productivity. Direct costs included hospital inpatient care, nursing home care, and medications, with inpatient care alone accounting for over $40 billion [4]. On average, individuals with diabetes incurred healthcare costs that were 2.4 times higher than those without diabetes, with a per capita expenditure of $13,243 compared to $2,560 for non-diabetic individuals [4]. Additionally, indirect costs stemmed from lost workdays, restricted activity, permanent disability, and premature mortality, emphasizing the broad economic impact of diabetes on productivity and healthcare systems [4]. These costs are expected to rise with increasing prevalence, aging populations, and associated comorbidities, highlighting the urgent need for effective prevention, management, and policy interventions to mitigate diabetes' global economic burden. 

T2DM is the leading cause of chronic kidney disease (CKD), and CKD affects approximately 10-15% of the world's population, and its prevalence has also been reported to be on the rise ​[5,6]. Diabetic kidney disease (DKD) is prevalent in approximately 40% of people with T2DM, and it significantly increases the risk of end-stage renal disease (ESRD) and cardiovascular mortality [7]. The comorbidity of these two conditions places a significant burden on healthcare systems, especially in low- and middle-income countries (LMICs), where both T2DM and CKD are rapidly increasing ​[5].

The bidirectional relationship between T2DM and CKD is a well-documented phenomenon. In patients with diabetes, prolonged hyperglycemia leads to glomerular damage, inflammation, and oxidative stress, which are key contributors to the development and progression of CKD [8]. CKD, in turn, exacerbates insulin resistance, resulting in poor glycemic control and the progression of diabetic complications [9]. CKD alters glucose metabolism due to decreased renal clearance of insulin and a reduced capacity for gluconeogenesis. Furthermore, CKD complicates the use of certain anti-diabetic medications, necessitating careful selection and dose adjustment to avoid adverse effects [10]. As CKD advances, it also impairs the excretion of medications used to manage T2DM, such as metformin and insulin, further complicating treatment [10]. 

The coexistence of CKD and T2DM significantly increases the risk of both microvascular and macrovascular complications. Microvascular complications, such as diabetic retinopathy, neuropathy, and nephropathy, are often exacerbated by CKD progression, leading to severe impairments, including blindness and ESRD [11]. Neuropathy, which affects both sensory and motor functions, reduces quality of life and becomes more challenging to manage as CKD advances [9]. Macrovascular complications, including coronary artery disease, peripheral artery disease (PAD), and cerebrovascular disease, are the leading causes of death in this population, with cardiovascular diseases (CVD) accounting for over 50% of mortality [10]. CKD amplifies traditional cardiovascular risk factors, such as hypertension, dyslipidemia, and inflammation, complicating the management of these risks. Additionally, reduced renal function interferes with medication metabolism, heightening the risk of adverse cardiovascular events [12]. CKD also accelerates atherosclerosis, further increasing the risk of myocardial infarction, stroke, and PAD, with many patients presenting with advanced disease at diagnosis [11]. The combination of poor glycemic control, impaired renal function, and accelerated atherosclerosis results in a poorer prognosis, increasing the likelihood of disability and death in these patients. We aimed to provide a comprehensive review of the pathophysiology and vascular complications arising from the coexistence of T2DM and CKD. By synthesizing current evidence, the review sought to elucidate the interconnected mechanisms that drive the progression of DKD and its associated microvascular and macrovascular complications. 

## Review

Pathophysiological links between type 2 diabetes mellitus (T2DM), chronic kidney disease (CKD), and vascular complications

**Figure 1:**
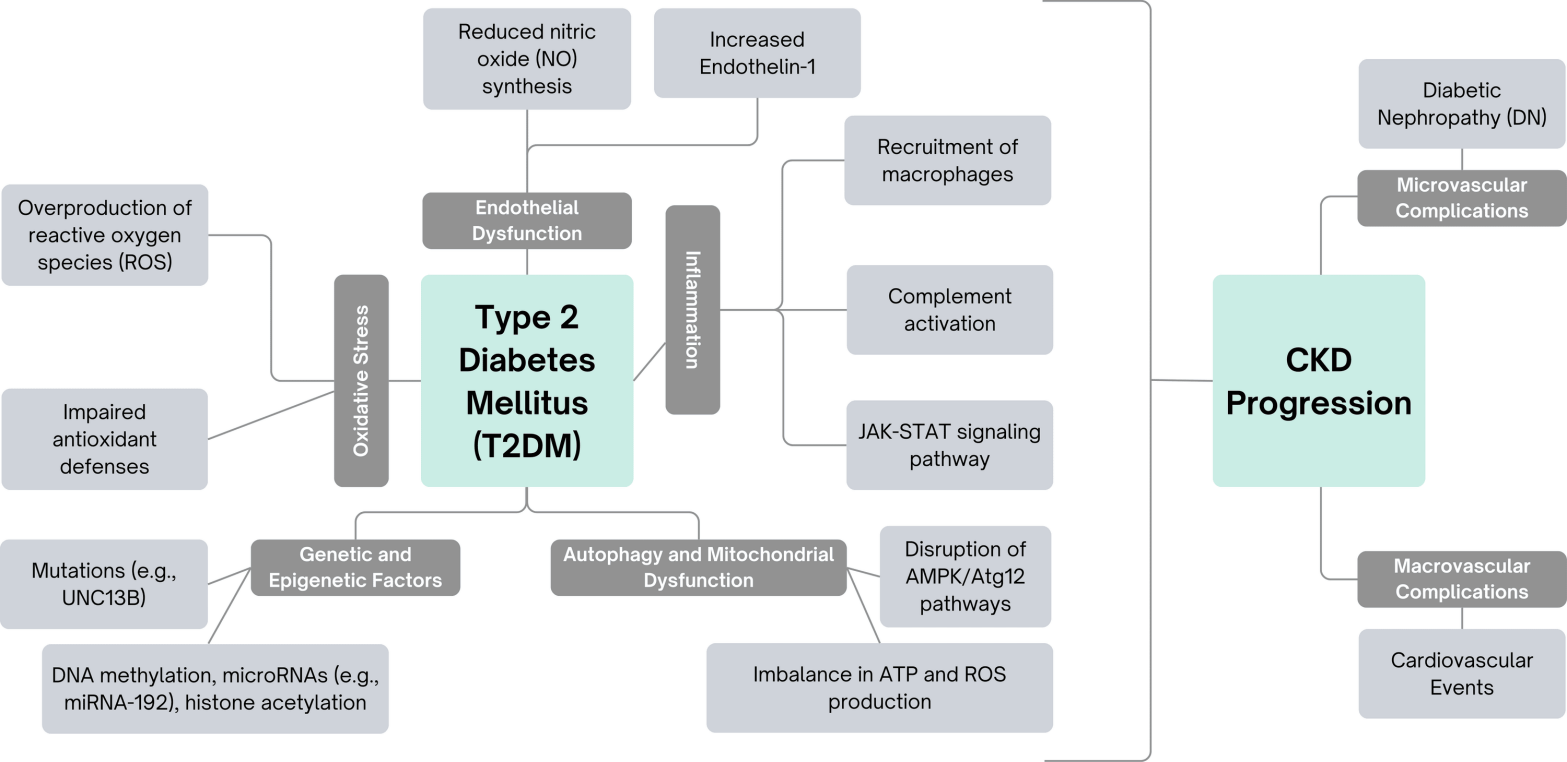
Schematic outlining the major pathways involved in the pathogenesis of DKD DKD - diabetic kidney disease; T2DM - type 2 diabetes mellitus; CKD - chronic kidney disease; ROS - reactive oxygen species; NO - nitric oxide; DN - diabetic nephropathy; AMPK - AMP-activated protein kinase; ATP - adenosine triphosphate, JAK-STAT - Janus Kinase-signal transducer and activator of transcription Image generated by authors

T2DM is a major contributor to CKD and vascular complications, impacting both microvascular and macrovascular systems (Figure 1). The progression of CKD in T2DM is primarily driven by three interconnected mechanisms: oxidative stress, chronic inflammation, and endothelial dysfunction [13]. These mechanisms work in a synergistic and bidirectional manner (Figure 1). For example, reactive oxygen species (ROS) cause cellular damage while simultaneously activating inflammatory pathways that recruit immune cells to the endothelium, further exacerbating oxidative stress [14]. This creates a self-reinforcing cycle that accelerates CKD progression and increases the risk of cardiovascular complications in diabetic patients [15]. Understanding these interrelated pathways offers crucial insights into the management of CKD and vascular complications in individuals with T2DM. 

Inflammation

Inflammation plays a central role in CKD, which is recognized as a systemic inflammatory condition with multiple contributing factors. It has been identified as a predictor of long-term CKD risk [8]. As kidney function deteriorates, proinflammatory mediators in the circulation progressively increase [8]. Inflammatory processes in CKD patients are driven by factors such as infections (e.g., periodontal disease), oxidative stress from the buildup of advanced glycation end products, metabolic acidosis, impaired cytokine clearance, insulin resistance, posttranslational modifications of circulating molecules like lipoproteins, and epigenetic influences [8].

Inflammation can drive the progression of DKD in T2DM. Persistent hyperglycemia activates nuclear factor kappa-light-chain-enhancer of activated B cells (NF-κB), inducing the production of pro-inflammatory cytokines such as tumor necrosis factor-alpha (TNF-α) and interleukin-6 (IL-6) [13]. These cytokines exacerbate endothelial damage, proteinuria, and fibrosis in the kidneys [16]. Hyperglycemia-induced stress activates the innate immune system, releasing chemokines and recruiting macrophages [13]. These macrophages form immune complexes that deposit in the kidneys, triggering complement activation and intensifying inflammation [13].

Ischemia associated with DKD further exacerbates inflammation by increasing neutrophil and macrophage infiltration, which accelerates renal injury [17]. The Janus kinase-signal transducer and activator of transcription (JAK-STAT) signaling pathway also contributes to inflammation in DKD, with increased expression in glomerular podocytes linked to disease progression [13,17]. Additionally, systemic inflammation is perpetuated by gut dysbiosis in T2DM and CKD, which compromises gut barrier integrity, facilitates bacterial translocation, and activates innate immune responses [17]. This complex interplay of inflammatory pathways drives proteinuria, vascular complications, and progressive renal damage in DKD. 

Oxidative stress

Oxidative stress has been suggested to play a main role in the pathogenesis of T2DM and its complications [13,14]. The study by Calabrese et al. [14] highlighted the critical role of oxidative stress and cellular stress responses in the progression of diabetic nephropathy in T2DM. The results showed that patients with diabetic nephropathy exhibited significantly elevated oxidative stress markers, including pentosidine, protein carbonyls, and lipid peroxidation products like F2-isoprostanes and 4-hydroxy-2-nonenal, compared to healthy controls (p<0.01) [14]. Increased expression of cellular stress proteins, such as Hsp60, Hsp70, and HO-1, correlated with oxidative damage and renal dysfunction, as indicated by proteinuria [14]. The study highlighted the interplay between oxidative stress, advanced glycation end-products, and stress responses, suggesting that targeting these pathways could offer therapeutic potential for managing diabetic nephropathy. 

The study by JhaJay et al. [18] "Diabetes and Kidney Disease: Role of Oxidative Stress" highlighted the critical role of oxidative stress in DKD, driven by an imbalance between reactive oxygen species (ROS) overproduction and inadequate antioxidant defenses. ROS, primarily generated by NADPH oxidases (NOXs) and mitochondrial dysfunction, contribute to glomerular injury, albuminuria, and fibrosis by inducing lipid peroxidation, protein modification, DNA damage, and activation of inflammatory pathways [18]. NOX4 is highlighted as a key enzyme promoting mesangial expansion and extracellular matrix accumulation in DKD, with experimental models showing that its inhibition reduces fibrosis, inflammation, and albuminuria [18]. The study also emphasized mitochondrial ROS's role in exacerbating kidney damage and discusses therapeutic strategies, including NOX inhibitors and antioxidants, which demonstrate potential in preclinical models to mitigate oxidative damage and slow DKD progression [18]. 

The intricate interplay of ROS production, metabolic dysregulation, and insufficient antioxidant defenses highlights oxidative stress as a central driver of DKD pathology. The overproduction of ROS activates intracellular signaling molecules, including cytokines and transcription factors, which perpetuate renal inflammation and fibrosis. While mitochondrial ROS also contribute to oxidative stress, NADPH oxidases are considered the primary long-term ROS source in DKD [13]. 

Endothelial dysfunction

Endothelial dysfunction is a key contributor to cardiovascular complications in diabetes mellitus, driven by mechanisms like reduced nitric oxide (NO) bioavailability, oxidative stress, and chronic inflammation [19]. Hyperglycemia stimulates reactive oxygen species (ROS) production via NADPH oxidase and mitochondrial pathways, inactivating NO and promoting vascular injury [18]. Inflammatory cytokines such as TNF-α and activation of protein kinase C (PKC) further exacerbate endothelial damage by amplifying oxidative stress and disrupting vascular homeostasis [13,19]. Clinically, this results in impaired vasodilation, arterial stiffness, and increased thrombotic risk, significantly elevating cardiovascular disease in diabetes patients [19]. Current therapeutic strategies, including statins, ACE inhibitors, and SIRT1 activators, aim to mitigate endothelial dysfunction, while emerging interventions targeting PKC and mitochondrial ROS show promise in addressing this critical aspect of diabetes-related vascular complications [20].

Normally, the endothelium regulates vascular tone, prevents thrombosis, and maintains an anti-inflammatory state. However, in T2DM, oxidative stress and chronic inflammation reduce NO production while increasing vasoconstrictive agents such as endothelin-1 [13]. This imbalance restricts renal perfusion and promotes systemic atherosclerosis, heightening the risk of cardiovascular events [21]. CKD further exacerbates endothelial dysfunction through mechanisms such as fluid overload and systemic inflammation, which impair NO synthesis and stiffen arteries, thereby contributing to both macrovascular (e.g., myocardial infarction, stroke) and microvascular (e.g., capillary damage) complications [21].

Endothelial cell injury significantly impacts the permeability of the glomerular filtration membrane, contributing to diabetic nephropathy (DN). Von Willebrand factor (vWF), primarily synthesized by endothelial cells, is a key biomarker of endothelial dysfunction [13]. Elevated plasma vWF levels have been observed in type 1 diabetes (T1D) and are significantly higher in DN patients compared to those without kidney disease, suggesting its role in the early diagnosis of DN [13,21].

Linden et al. [22] study revealed a strong association between advanced glycation end products (AGEs) and endothelial dysfunction in CKD. Elevated AGE levels were inversely correlated with glomerular filtration rate (GFR) and endothelial function markers, such as postocclusive reactive hyperemia and thermal hyperemia [22]. Additionally, AGEs were linked to increased receptor for AGE (RAGE) expression in peripheral blood mononuclear cells. AGE-rich serum fractions from CKD patients suppressed endothelial nitric oxide synthase (eNOS) expression in human aortic endothelial cells, a critical factor for vascular health, an effect that was reversed with RAGE blockade [22]. These findings suggest that AGE-induced activation of RAGE plays a pivotal role in endothelial dysfunction in CKD, providing a potential therapeutic target for reducing cardiovascular complications in this population [22].

Endothelial dysfunction is a critical factor in the progression of hypertension and diabetes mellitus (DM), driven by chronic inflammation and oxidative stress. Key inflammatory mediators, TNF-α and IL-6, upregulate adhesion molecules like ICAM-1 and VCAM-1, exacerbating vascular inflammation and stiffness. Chronic hyperglycemia and elevated blood pressure reduce nitric oxide availability through eNOS uncoupling, perpetuating endothelial damage [23,24]. In kidney transplantation, ED persists due to uremic toxins and inflammatory cytokines despite partial vascular recovery post-transplantation. Targeted therapies, such as IL-1β inhibitors, have shown potential in mitigating inflammation and improving endothelial function, offering promising strategies to reduce cardiovascular risks in HTN and DM [25].

Genetic and epigenetic regulations 

Genetic and epigenetic factors play a crucial role in the progression of DKD. Epigenetic changes, including DNA methylation, non-coding RNAs like miRNA-192, and histone modifications, are influenced by high blood sugar inflammation [26,27]. These changes promote kidney damage by increasing fibrosis and extracellular matrix buildup, as seen with the hypermethylation of genes like RASALI and the activity of miRNA-192 [26,27]. The antioxidant defense system, regulated by Nrf2, is weakened in DKD, making the kidneys more vulnerable to oxidative stress. Podocyte autophagy, essential for maintaining healthy kidney cells, is disrupted by factors like β-arrestins and excessive mTOR activity [28]. Mitochondrial dysfunction further contributes by reducing energy production and activating inflammatory pathways. Restoring balance through interventions like exercise or medications that boost AMPK activity can improve mitochondrial function and enhance autophagy [28,29].

Common microvascular complications in T2DM and CKD

**Figure 2:**
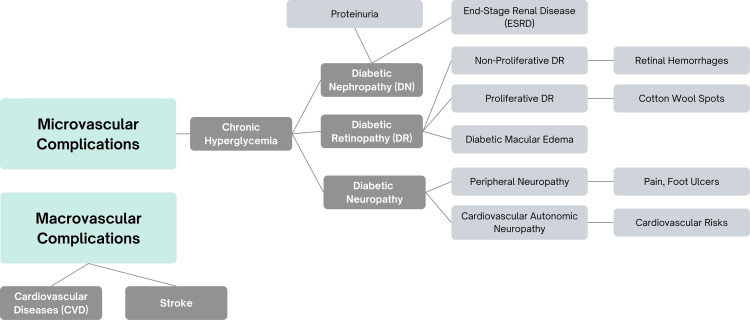
Common microvascular and macrovascular complications of T2DM in CKD T2DM - type 2 diabetes mellitus; CKD - chronic kidney disease; DN - diabetic nephropathy; DR - diabetic retinopathy; ESRD - end-stage renal disease; CVD - cardiovascular diseases; DR - diabetic retinopathy

Common microvascular complications in T2DM and CKD include diabetic nephropathy, diabetic retinopathy, and diabetic neuropathy (Figure 2). Diabetic nephropathy is characterized by albuminuria, declining kidney function, and eventual progression to ESRD [30,31]. Diabetic retinopathy affects the small blood vessels in the retina, leading to vision issues such as blurred vision, macular edema, or even blindness if untreated. Diabetic neuropathy causes nerve damage, resulting in symptoms like tingling, pain, or numbness, particularly in the extremities, as well as autonomic dysfunction affecting various organ systems (Figure 2). These complications significantly impair quality of life and require early detection and management to reduce long-term consequences.

CKD-diabetic retinopathy

Diabetic retinopathy (DR) is one of the leading causes of blindness in individuals with diabetes [32]. It results from damage to the blood vessels of the retina, primarily caused by chronic hyperglycemia, inflammation, and oxidative stress [32]. DR presents in various forms, including severe non-proliferative diabetic retinopathy (NPDR), proliferative diabetic retinopathy (PDR), and diabetic macular edema [32,33]. The prevalence of DR increases with the duration of diabetes, ranging from 28.8% in individuals with diabetes for less than five years to 77.8% in those with diabetes for 15 or more years. Proliferative DR, a more severe form, affects 2.0% of those with diabetes for less than five years and 15.5% of those with diabetes for 15 or more years [33].

Patients with both T2DM and CKD are at an even higher risk for DR due to overlapping risk factors such as hypertension, dyslipidemia, and persistent hyperglycemia [11]. Clinical findings of DR often include hemorrhages and cotton wool spots, which can be assessed using fundus fluorescein angiography (FFA) [11]. Univariate regression analyses have shown that advanced DR in older patients with T2DM correlates with a higher prevalence of advanced CKD, even after accounting for factors such as age, sex, duration of diabetes, and comorbidities like hypertension and cardiovascular disease [34]. Early detection and management of DR through regular screening are essential for preventing severe visual impairment. Effective treatment options for PDR, such as panretinal photocoagulation and intravitreal injections of ranibizumab, have shown promise in reducing disease progression and improving outcomes [34]. Achieving better glycemic control has also been associated with a lower prevalence of DR, emphasizing the importance of tight metabolic regulation [34].

The study by Lin et al. [35] examined the impact of DR on CKD progression in diabetic patients, finding a significant association between DR severity and worsening renal function. Patients with DR had poorer renal function, lower estimated glomerular filtration rates (eGFR), and higher rates of proteinuria, hypertension, and anemia compared to those without DR [35]. CKD progression was more frequent in the DR group (21.67%) than in the non-DR group (13.62%), with the risk especially high in advanced CKD stages (3b-5). Proliferative DR posed a greater risk for CKD progression compared to non-proliferative DR [35]. The study emphasized the shared mechanisms between DR and CKD, such as inflammation and microvascular damage, highlighting the importance of early detection and management of DR to slow CKD progression, particularly in advanced disease stages [35].

A recent Tongren Health Care Study explored the relationship between DR and CKD in patients with T2DM. Among 5,103 participants with T2DM, the prevalence of DR was 12.8%, while CKD was observed at 13.3% [36]. DR was detected in 21.0% of patients with CKD, and CKD was present in 20.9% of those with DR [36]. DR prevalence was significantly associated with higher rates of albuminuria and reduced eGFR (p<0.05). Factors linked to CKD rather than DR included older age, higher body mass index, elevated triglycerides, and hypertension (OR=4.47 for reduced eGFR, p=0.005) [36]. Conversely, higher blood glucose levels were associated with a greater likelihood of DR. These findings suggest distinct risk profiles for DR and CKD in T2DM, emphasizing the need for tailored screening and management strategies [36]. The coexistence of DKD and DR represents a significant clinical challenge, as these common microvascular complications often share overlapping risk factors and contribute to increased disease burden in individuals with diabetes. 

Reis et al.'s [37] study examined clinical features associated with the concurrent presence of DKD and DR in patients with T1D and T2DM. Among 517 patients with DKD, 236 (45.6%) had DR, with 83.8% of the cohort comprising individuals with T2DM. Patients with T2DM and DR were more likely to use insulin (OR=3.63, 95% CI 1.89-7.00), have a longer duration of diabetes (OR=1.04 per year, 95% CI 1.02-1.07), and exhibit higher systolic blood pressure (OR=1.01 per mmHg, 95% CI 1.00-1.02) [37]. No significant predictors of DR were identified in patients with T1D. The study also noted that the prevalence of DR in this cohort was higher than global averages, potentially reflecting more advanced disease due to prolonged diabetes duration and greater use of insulin [37]. These findings highlight the importance of monitoring patients with DKD who exhibit these risk factors for the development of DR to enable early intervention.

Lee et al. [38] study explored the association between DR severity and CKD progression in older patients with T2DM. Among 116 participants with CKD stage ≥3, patients with more severe DR showed significant deterioration in renal function [38]. Proliferative DR (PDR) was associated with the highest prevalence of advanced CKD (86%), compared to 36% in those without DR. Key markers of renal function, such as eGFR, declined with increasing DR severity, while macroalbuminuria was significantly more common in patients with PDR [38]. After adjusting for confounding factors like age, diabetes duration, and insulin use, the odds of advanced CKD were approximately 4.6 times higher in non-proliferative DR (NPDR) and 11.8 times higher in PDR compared to those without DR [38]. These findings underscore the importance of routine ophthalmologic and renal evaluations in older diabetic patients to mitigate CKD progression, particularly as DR severity appears to be a strong predictor of renal dysfunction. Despite its retrospective design and single-center setting, the study highlights the need for integrated management of diabetic complications in this population.

CKD-diabetic neuropathy

Diabetic neuropathy is a common complication of both T1D and T2DM, characterized by peripheral nerve dysfunction manifesting as pain, numbness, tingling, and weakness [39]. It can affect peripheral, autonomic, and cranial nerves and is thought to result from prolonged hyperglycemia, metabolic imbalances, and oxidative stress [39]. Patients with stage 4-5 CKD due to diabetic nephropathy often experience multiple neurological complications, including diabetic peripheral neuropathy (DPN) and cardiovascular autonomic neuropathy (CAN) [39]. These conditions significantly increase morbidity and mortality while negatively impacting the quality of life of individuals with advanced CKD [39,40]. Addressing complications such as CAN and DPN is especially critical in late-stage CKD, where these conditions exacerbate the already substantial health burden [30,31]. Together, DR and neuropathy highlight the complex interplay between T2DM and CKD, where shared pathophysiological mechanisms and overlapping risk factors necessitate a comprehensive approach to patient care. Early diagnosis, meticulous glycemic control, and targeted interventions remain key to mitigating the impact of these debilitating complications.

The significant burden of diabetic neuropathy in patients with advanced CKD and those on dialysis is highlighted by the high prevalence of CAN and DPN [30]. CAN affects up to 62% of dialysis patients, contributing to increased risks of sudden cardiac death, while DPN, often presenting with neuropathic pain, affects nearly half of these patients and leads to high rates of lower limb amputation and mortality [30]. Hyperglycemia, uremia, and oxidative stress are central to the pathogenesis, with sympathetic overactivity worsening CAN outcomes. Strict glycemic control remains the primary strategy for slowing neuropathy progression, particularly in T1D, although evidence in T2DM is less robust. Pain management requires careful selection and adjustment of therapies like gabapentin and duloxetine to accommodate CKD-related limitations [30]. 

A study analyzed the association of DPN with DKD and CVD in a population of 184 patients using the Michigan Neuropathy Screening Instrument (MNSI); 37.5% of participants were diagnosed with DPN [41]. The analysis revealed significant associations between DPN and DKD (26% in the DPN group vs. 3.5% without, OR=6.38, p=0.0034) and CVD (49.3% in the DPN group vs. 20.1% without, p=0.00006). DPN patients had higher mean age (68.7 ± 11.6 years vs. 50.8 ± 17.1 years) and longer diabetes duration (15.75 ± 10.8 years vs. 11.29 ± 9.24 years) [41]. No significant differences were observed in HbA1c levels or diabetic retinopathy prevalence between groups [41]. The findings underline the importance of screening for additional complications when one is identified, highlighting the interconnected nature of microvascular and macrovascular diabetes-related complications. The authors called for broader research to validate these associations and suggest individualized glycemic management strategies to mitigate risks [41].

Yang et al. [42] study investigated the relationship between markers of DKD and DPN in T2DM patients. Among 471 participants, 71.1% were diagnosed with DPN, which was associated with older age, longer diabetes duration, and poorer glycemic control. The study identified a progressive increase in DPN prevalence with worsening kidney function, rising from early to advanced CKD stages [42]. Among the renal injury markers studied, urinary N-acetyl-β-D-glucosaminidase/creatinine ratio (NAG/Cr) emerged as an independent predictor of DPN, highlighting its potential role in early detection [42]. The findings underline the interconnected nature of diabetes complications and emphasize the importance of monitoring renal and nerve health to improve patient outcomes [42].

Peripheral neuropathy (PN) is a common complication in patients with CKD, particularly those with coexisting diabetes, and its impact on long-term outcomes warrants further investigation. The relationship between peripheral neuropathy (PN) and mortality in patients with CKD using NHANES data found that PN is more prevalent in CKD patients compared to those without CKD and is further exacerbated by diabetes [43]. Over 14 years of follow-up, PN emerged as an independent risk factor for increased all-cause and cardiovascular mortality in CKD patients, suggesting that the presence of PN reflects systemic complications that negatively impact survival [43]. The study emphasizes the need to address PN as a key target in managing CKD-related complications to improve outcomes and reduce mortality risks. 

CKD-diabetic nephropathy

Diabetic nephropathy (DN) is a chronic complication of diabetes characterized by elevated urinary albumin excretion and a progressive decline in kidney function [44]. It results from damage to the glomerular capillaries, which normally filter waste and excess substances from the blood [44]. This condition typically arises due to the interplay between metabolic and hemodynamic factors, which activate specific pathways leading to kidney injury. Studies have shown that intensive glucose control can mitigate the progression of DN [44]. In the early stages of diabetic nephropathy, hyperfiltration occurs as a result of hemodynamic changes, primarily driven by hyperglycemia-induced vasodilation of the afferent arterioles, leading to increased glomerular filtration rate [44]. Over time, this process causes structural damage to the glomeruli, progressing from microalbuminuria to macroalbuminuria, which reflects worsening kidney function [44].

Critical factors in the development of DN include metabolic changes, hemodynamic alterations, inflammation, and genetic predisposition. Hyperglycemia-induced metabolic changes contribute to the formation of advanced glycation end-products (AGEs) and activation of the polyol pathway, leading to oxidative stress and tissue damage. These metabolic alterations are closely linked to structural abnormalities in the kidney, such as the thickening of the glomerular basement membrane, which impairs normal kidney function. Other pathological changes include the formation of microaneurysms and mesangial nodules (Kimmelstiel-Wilson bodies), further exacerbating renal dysfunction by disrupting glomerular architecture and filtration [45,46].

The study by Liu et al. [47] explored the differentiation between DKD and CKD using clinical and biochemical markers from 1,726 inpatients. Among these, 319 had DKD, and 1407 had CKD. DKD patients were predominantly female (64.6%) compared to CKD (55.4%), with women showing a higher susceptibility (OR=2.234). DKD patients exhibited higher diastolic blood pressure, fasting glucose, total cholesterol, and urinary volume, with elevated total cholesterol (TCHO) increasing DKD risk threefold. Conversely, lower vitamin B12 levels were more common in CKD patients, serving as a distinguishing factor [47]. A discriminant formula incorporating 11 variables, such as eGFR and fasting glucose, demonstrated high accuracy (94.1 %) in differentiating between CKD and DKD. The findings suggest that DKD patients have milder eGFR reductions compared to CKD, while elevated cholesterol and abnormal urinary markers are significant DKD indicators [47]. The study concluded that a combination of clinical and biochemical parameters can effectively differentiate the two conditions, aiding early diagnosis and tailored management [47].

Diabetic nephropathy (DN) and DKD are the leading causes of CKD and end-stage kidney disease (ESKD) globally. However, while 30-50% of ESKD cases in the U.S. are attributable to diabetes, only 30-40% of diabetic patients develop nephropathy. Genetic predisposition, socioeconomic factors, and pathophysiological changes are critical contributors to DKD progression. Proteinuria remains a pivotal predictor of disease progression, with annual eGFR loss rates reduced from 7-12 mL/min to 3-6 mL/min with renin-angiotensin system (RAS) inhibitors [48].

Disease progression, marked by declining eGFR and increasing serum creatinine and blood urea nitrogen levels, was particularly pronounced in patients with diabetes duration over 10 years, with 60% of these patients reaching stage 3 CKD [49]. ON significantly impaired HRQOL, with patients in advanced stages experiencing worse physical and mental health outcomes and more severe symptoms [49]. Risk analysis identified obesity (OR 7.08) and prolonged diabetes duration (OR 6.48) as the strongest predictors of ON progression [49]. The study emphasized the critical importance of early screening for proteinuria and proactive management of modifiable risk factors like obesity and hypertension to improve outcomes and slow disease progression, recommending routine urinalysis as a key tool in diabetes care [49].

Common macrovascular complications

Stroke

CKD increases stroke risk across all stages, with severe proteinuria, such as in nephrotic syndrome, independently exacerbating this risk. In most patients with kidney disease, the elevated stroke risk is primarily associated with atrial fibrillation (AF) and common atherosclerotic risk factors. Additionally, hemodialysis-specific issues, such as cerebral hypoperfusion, contribute to further stroke risk in this population. Patients with CKD also experience worse stroke outcomes, including greater severity, higher recurrence, and increased mortality rates. The underrepresentation of CKD patients in clinical trials results in significant gaps in evidence-based stroke care for this group [50]. 

The study by Kaze et al. [51] examined the link between kidney function abnormalities and stroke risk in adults with T2DM using data from 9,170 participants in the ACCORD trial. Over a median follow-up of 4.9 years, severe albuminuria (UACR 2:300 mg/g} was associated with 2.29-fold increased stroke risk, while reduced eGFR (<60 mL/min/1.73 m2} increased the risk by 1.50-fold. Higher CKD stages corresponded to greater stroke risk, with stage G3 (eGFR 30-59 ml/min/1.73 m2) doubling the hazard (HR 2.03) compared to those without CKD [51]. The study highlighted albuminuria in patients with at least moderately reduced eGFR and reduced eGFR as independent predictors of stroke, reflecting systemic vascular dysfunction and inflammation [51]. The findings emphasized the importance of early identification and management of CKD in diabetic patients to mitigate stroke risk and improve cardiovascular outcomes [51].

Gomes et al. [52] assessed stroke awareness among 197 CKD patients on hemodialysis in northeastern Brazil, revealing significant knowledge gaps about stroke symptoms, risk factors, and emergency response. Only 29.9% could identify one symptom and risk factor, and less than 10% demonstrated ideal decision-making capacity regarding stroke emergencies [52]. These findings underscore the importance of balancing the risks of bleeding and embolism in CKD patients, particularly given the increased bleeding risk observed in this population. Targeted health education campaigns are essential to improve stroke recognition and response in underserved regions, ultimately enhancing outcomes for high-risk CKD patients.

Cardiovascular diseases

Atherosclerosis, driven by chronic inflammation and endothelial injury, is the central pathological mechanism in macrovascular complications of diabetes [53-56]. Hyperglycemia promotes the accumulation of oxidized lipids in arterial walls, leading to the formation of foam cells, smooth muscle proliferation, and fibrous plaques that may rupture, causing acute vascular infarction [53,57]. Diabetes also increases platelet aggregation, reduces nitric oxide (NO) availability, and impairs fibrinolysis, further elevating the risk of cardiovascular events [57]. These mechanisms make diabetes a significant risk factor for CVD, stroke, and coronary artery disease (CAD) [57]. Studies have shown that the risk of myocardial infarction (MI) in diabetic patients is equivalent to that of non-diabetic patients with prior MI, leading to the classification of diabetes as a CAD risk equivalent [53].

Diabetes mellitus, hypertension, and chronic kidney disease significantly contribute to systemic inflammation and endothelial dysfunction, both of which are critical in the pathogenesis of atrial fibrillation (AF) and coronary artery disease (CAD). Poorly controlled DM leads to structural alterations in the atrial myocardium, including fibrosis and hypoxemia, driven by inflammation, oxidative stress, and advanced glycation end products (AGEs), which collectively increase AF risk [54,55]. HTN exacerbates this by inducing atrial fibrosis through renin-angiotensin-aldosterone system activation and proinflammatory cytokines, further promoting AF via ectopic firing and re-entry mechanisms [54]. CAD acts as a common ground for these conditions, as ischemia triggers atrial remodeling and local inflammation, creating a cycle of fibrosis and prolonged conduction times​​ [54,55]. Conversely, AF worsens CAD by inducing endothelial dysfunction and systemic inflammation, driven by reduced nitric oxide availability and increased von Willebrand factor expression, which lead to atherosclerotic plaque instability and ischemia. Managing this interconnected pathology requires aggressive control of shared risk factors, anti-inflammatory therapies like IL-1β inhibitors, and lifestyle interventions targeting glycemic and blood pressure control​ [54,55,56].

Oliveira et al. [20] study assessed cardiovascular risk factors among CKD patients under conservative treatment, revealing a high prevalence of risk factors such as hypertension (87.2%), diabetes (53.5%), physical inactivity (62.2%), and elevated body mass index (BMI), waist circumference (WC), and body fat percentage (BF%) [20]. Overweight was noted in 82.4% of adults under 60 and 60.6% of older adults, with significantly high WC in females (91.9%) and males (64.3%) [20]. Biochemical findings included elevated uric acid in 73.3% of participants and high glycated hemoglobin (HbA1C) levels in 72.7% of diabetics, indicating poor glycemic control [20]. Using the Framingham score, 43% were classified as low CVD risk, while 35.5% and 21.5% were at medium and high risk, respectively [20]. These findings emphasize the need for multidisciplinary interventions targeting modifiable risk factors to reduce CVD mortality among CKD patients.

CKD and ESRD, treated with conventional dialysis, are strongly associated with left ventricular hypertrophy (LVH), intermyocardial fibrosis, and capillary loss, which increase the risk of sudden cardiac death and heart failure [58]. These abnormalities, detectable through cardiac MRI or echocardiography, result from factors such as arterial pressure, intravascular volume, anemia, and molecular mediators [58]. LVH affects approximately one-third of CKD patients, rising to 70-80% in those with ESKD [59]. This condition is an independent survival predictor, even in early CKD stages. The development of LVH is attributed to three primary mechanisms: 1) afterload-related factors such as arterial stiffness, systemic arterial resistance, and systolic hypertension, initially leading to concentric LVH, and eventually eccentric hypertrophy, dilatation, and reduced ejection fraction; 2) preload-related factors, including intravascular volume expansion causing volume overload and asymmetric remodeling; and 3) non-afterload, non-preload factors, including intracellular signaling pathways, metabolic changes, and reduced fatty acid oxidation [58]. Myocardial fibrosis, independent of LVH, involves diffuse collagen deposition between capillaries and cardiomyocytes, contributing to maladaptive ventricular remodeling and heart enlargement [59].

Additionally, CKD is strongly associated with aortic and mitral valve disease, which significantly impacts patient outcomes [60]. The study by Wang et al. [60] highlighted the significant impact of nephropathy in diabetic patients on the development of cardiac valve calcification (CVC), a major predictor of cardiovascular and all-cause mortality in dialysis patients. Nephropathy contributes to mineral metabolism abnormalities, including hyperphosphatemia, hypercalcemia, and secondary hyperparathyroidism, which drive vascular calcification, affecting up to 90% of stage 5 CKD patients on dialysis [60]. CVC exacerbates cardiovascular risks by increasing arterial stiffness, reducing vascular compliance, and promoting left ventricular hypertrophy [60]. The study found CVC-associated hazard ratios of 2.81 for cardiovascular mortality and 1.73 for all-cause mortality, with higher risks in Asian populations and peritoneal dialysis patients. These findings emphasize the need for early identification and management of mineral imbalances and systemic inflammation to improve outcomes in diabetic nephropathy patients undergoing dialysis [60]. In addition, early CKD stages (1-3) have been linked to increased calcification of heart valves and coronary arteries, with valve calcification affecting up to 88-99% of patients in CKD stage 5. This rate rises from 40% in stage 3 CKD, with valve destruction occurring 10 times more frequently in CKD patients compared to those without CKD [59,61]. 

Valvular calcification (VC) is a significant but often underappreciated complication of CKD, contributing to increased mortality and distinct clinical outcomes compared to vascular calcification [62]. CKD patients are disproportionately affected, with aortic and mitral valve calcification prevalence ranging from 25% to 59% and calcifications occurring 10-20 years earlier than in the general population [62]. In dialysis patients, the annual incidence of aortic valve calcification is approximately 3.3%. These calcifications are closely linked to cardiovascular complications, including ischemic heart disease, arrhythmias, sudden cardiac death, heart failure, and stroke, which together account for over 50% of deaths in advanced CKD [62]. Urena-Torres [62] emphasized the need for greater attention to VC in CKD due to its unique pathophysiological mechanisms, earlier onset, and significant impact on survival, highlighting the potential for targeted therapeutic interventions to mitigate its clinical consequences. The progression of valve disease is accelerated by factors such as diabetes, hypertension, hyperlipidemia, anemia, infections, malnutrition, and mineral metabolism abnormalities like hypercalcemia, hyperphosphatemia, and hyperparathyroidism [62]. 

Emerging therapies in diabetic kidney disease

For many years, RAAS blockers have been the primary treatment for diabetic nephropathy, forming the foundation for slowing disease progression. Recent innovations have introduced new therapeutic classes, including SGLT2 inhibitors and nonsteroidal mineralocorticoid receptor antagonists (MRAs), which provide additional renal and cardiovascular benefits. According to Kim et al. [63], emerging medications with strong evidence from large clinical trials include finerenone, semaglutide, liraglutide, DPP-4 inhibitors, and the NRF2 activator bardoxolone. Additionally, several novel agents are in development, focusing on oxidative stress and proinflammatory cytokines/chemokines [63]. These therapies, which have shown promising outcomes in preclinical studies, could pave the way for new strategies for managing diabetic kidney disease in the future.

Emerging therapies for diabetic kidney disease (DKD) focus on addressing its complex and multifaceted pathophysiology through innovative treatment strategies. Sodium-glucose cotransporter-2 inhibitors (SGLT2i), such as empagliflozin and dapagliflozin, have demonstrated significant renal and cardiovascular benefits, including reducing albuminuria, slowing the progression of CKD, and decreasing cardiovascular events by targeting glucose reabsorption and intraglomerular pressure [64,65]. Nonsteroidal mineralocorticoid receptor antagonists (nsMRAs), such as finerenone, are emerging as effective therapies for mitigating inflammation and fibrosis, critical drivers of DKD progression. These agents provide additional benefits when combined with established therapies, such as SGLT2i and renin-angiotensin-aldosterone system (RAAS) inhibitors, as evidenced in the FIDELIO-DKD and FIGARO-DKD trials [65]. In addition, these agents have been shown to delay DKD progression by up to 5.5 years and improve cardiovascular event-free survival by 3.2 years compared to conventional therapies [64].

Glucagon-like peptide-1 receptor agonists (GLP-1 RAs), including liraglutide and semaglutide, are also gaining prominence for their renal and cardiovascular protective effects, particularly in reducing albuminuria and aiding glycemic control. Experimental therapies, such as endothelin receptor antagonists and antifibrotic agents like pirfenidone, are being investigated, although their clinical use remains limited [66]. Furthermore, advancements in personalized medicine, such as biomarker-driven therapy, hold promise for refining treatment strategies and enabling tailored approaches to mitigate DKD progression and associated complications. This integrative, "pillar-based" approach to treatment - combining therapies with complementary mechanisms of action - not only addresses residual risks but also offers the potential for sustained and early benefits in CKD and cardiovascular management [64,65].

Practice recommendations

Early Screening and Monitoring

Regular and comprehensive screening for diabetes-related complications should be a cornerstone of care for patients with CKD and diabetes. Early identification of microvascular complications, such as diabetic nephropathy and retinopathy, is critical to prevent progression. Annual evaluations, including urine albumin-to-creatinine ratio (UACR), estimated glomerular filtration rate (eGFR), and ophthalmologic exams, are essential for early detection. In patients with advanced CKD or those on dialysis, periodic screening for neuropathy, including cardiovascular autonomic neuropathy, is recommended to identify high-risk individuals and initiate timely interventions. Furthermore, stratifying patients by their risk levels using validated tools or biomarkers, such as N-acetyl-β-D-glucosaminidase (NAG) for renal injury or advanced glycation end-products (AGEs) for endothelial dysfunction, can enhance precision in managing complications. 

Tools like UACR, eGFR, and biomarkers such as NAG and AGEs play a vital role in early detection and risk stratification, though applying these screening methods in low-resource settings remains a challenge. Limited access to laboratory facilities, high costs of advanced biomarker assays, and shortages of trained healthcare professionals are significant barriers in these regions. Additionally, awareness about the importance of early detection of CKD and diabetes may be limited among healthcare providers and patients, further delaying interventions. Addressing these challenges requires innovative approaches, such as integrating low-cost point-of-care testing for UACR and eGFR into primary care settings, training community health workers to perform basic screenings, and increasing public health education to enhance awareness. Partnerships with public health organizations and investments in scalable, cost-effective diagnostic technologies could help bridge these gaps, ensuring that early screening and monitoring become accessible to a broader population.

Integrated Management and Lifestyle Interventions

We recommend a multidisciplinary approach, incorporating endocrinologists, nephrologists, cardiologists, and dietitians as essential for the holistic management of CKD and diabetes. Glycemic control remains paramount; however, therapy should be tailored to account for altered drug pharmacokinetics in CKD. Lifestyle interventions, including personalized diet plans to limit sodium and protein intake, should be coupled with physical activity tailored to the patient's cardiovascular and renal status. Addressing modifiable risk factors like hypertension, dyslipidemia, and obesity with aggressive treatment strategies, including ACE inhibitors or ARBs and statins, is critical to reducing the burden of macrovascular complications. Additionally, the role of mineralocorticoid receptor antagonists (MRAs), both steroidal and nonsteroidal should be considered in managing fluid overload and controlling blood pressure. Patient education on medication adherence, symptom monitoring, and the importance of routine follow-ups is key to improving outcomes and quality of life. 

While a multidisciplinary approach and lifestyle interventions are crucial, addressing barriers to medication adherence is equally important, especially in underserved populations. Factors such as cost, access to healthcare, and lack of transportation often impede adherence to prescribed therapies. Addressing these barriers requires a multifaceted approach, including simplifying medication regimens, offering generic or subsidized alternatives to reduce costs, and leveraging telemedicine or mobile health platforms to improve access. Patient education programs tailored to individual needs and cultural contexts can also play a key role in enhancing understanding and adherence. Building strong patient-provider relationships and incorporating community health workers into care teams can further support medication adherence and improve overall outcomes.

Limitations of the study

The primary limitation of this study lies in its narrative review approach, which does not follow a systematic methodology for literature search and selection. This methodology may introduce selection bias. Additionally, the study does not address how the discussed mechanisms might differ across various populations, such as pediatric patients, elderly individuals, or those with comorbidities, which restricts the applicability of the conclusions to these groups. Furthermore, the article focuses predominantly on the pathophysiology and vascular complications of diabetes in chronic kidney disease (CKD), limiting the discussion of broader topics, such as the impact of emerging treatment strategies or non-diabetes-related causes of CKD. These constraints are inherent to the narrative review format but provide a foundation for future research that could adopt a more systematic approach to address these gaps.

## Conclusions

The intersection of type 2 diabetes mellitus (T2DM) and chronic kidney disease (CKD) creates a complex interplay of pathophysiological mechanisms that exacerbate both microvascular and macrovascular complications. Inflammatory pathways, oxidative stress, and endothelial dysfunction emerge as key drivers of this bidirectional relationship, amplifying the risks of diabetic nephropathy, retinopathy, neuropathy, and cardiovascular disease. These complications not only degrade patient quality of life but also significantly burden healthcare systems, particularly in low- and middle-income countries.

Integrated management approaches, encompassing early detection, personalized treatment regimens, and targeted interventions, are essential for mitigating the progression of diabetic kidney disease (DKD) and its vascular complications. Lifestyle modifications, multidisciplinary collaboration, and novel therapeutic strategies hold promise in improving outcomes and reducing morbidity and mortality. Further research is recommended to refine diagnostic tools, elucidate genetic and epigenetic contributors, and develop innovative treatments to address the challenges posed by T2DM and CKD. Ultimately, a paradigm shift toward proactive and patient-centered care is crucial for addressing this global health challenge, with a focus on preventing disease progression, optimizing treatment outcomes, and enhancing patient quality of life. Emerging therapies for DKD, including SGLT2 inhibitors, nonsteroidal MRAs, and GLP-1 receptor agonists, are transforming treatment by offering significant renal and cardiovascular benefits. Experimental approaches and personalized medicine further enhance management strategies, highlighting the need for ongoing research to address residual risks and refine therapies.
